# Antioxidant and Angiotensin I-Converting Enzyme (ACE) Inhibitory Peptides Obtained from Alcalase Protein Hydrolysate Fractions of Hemp (*Cannabis sativa* L.) Bran

**DOI:** 10.1021/acs.jafc.1c01487

**Published:** 2021-08-06

**Authors:** Seyedeh P. Samaei, Serena Martini, Davide Tagliazucchi, Andrea Gianotti, Elena Babini

**Affiliations:** † Department of Agricultural and Food Sciences, Alma Mater Studiorum, University of Bologna, Piazza Goidanich 60, 47521 Cesena, Italy; ‡ Department of Life Sciences (DSV), University of Modena and Reggio Emilia, Via Amendola 2, 42122 Reggio Emilia, Italy; § CIRI (Interdepartmental Centre of Agri-Food Industrial Research), Alma Mater Studiorum, University of Bologna, Via Quinto Bucci 336, 47521 Cesena (FC), Italy

**Keywords:** food byproduct, protein hydrolysates, antihypertensive peptides, antioxidant peptides, high-resolution mass spectrometry

## Abstract

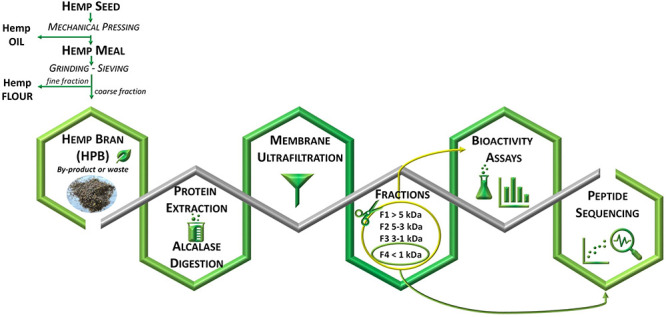

Proteins from hemp bran (HPB), a byproduct of the hemp seed food-processing chain, were chemically extracted, hydrolyzed by Alcalase, and separated by membrane ultrafiltration into four fractions (MW <1, 1–3, 3–5, and >5 kDa). The antioxidant and antihypertensive properties of the initial extract and the fractions were evaluated by *in vitro* assays for their ability to scavenge radical species, bind with metal ions, reduce ferric ions, and inhibit angiotensin-converting enzyme (ACE) activity. Bioactive peptides were identified by high-resolution mass spectrometry and sequence comparison with BIOPEP and BioPep DB databases. The hydrolysate was strongly antioxidant and ACE-inhibiting; the most bioactive peptides were further concentrated by ultrafiltration. Of the 239 peptides identified, 47 (12 antioxidant and 35 ACE-inhibitory) exhibited structural features correlated with the specific bioactivity. These results highlight the promise of hydrolysate and size-based HPB fractions as natural functional ingredients for the food or pharmaceutical industry.

## Introduction

In the last few years, the recovery of proteins from agricultural and agro-food industry waste byproducts has garnered more attention, as improving the sustainability of the food chain has become more urgent. Aside from their nutritional properties, these proteins have additional value when transformed into bioactive hydrolysates, which may be beneficial for human health and have potential food and pharmaceutical applications.^1^ A classic approach for the preparation of these hydrolysates is the enzyme-catalyzed treatment of extracted proteins. The proteolytic reaction breaks down the primary sequence, producing mixtures of peptides with many positive health effects (antioxidant, antihypertensive, anti-inflammatory, anticancer, and antimicrobial, among others).^2,3^ The bioactivity of the crude hydrolysates can be enhanced by chemically or physically based downstream separation processes, which concentrate the bioactive peptides in mixtures of lower complexity.^4−15^ Purified peptides from these mixtures could provide even higher potency and greater specificity, but the costs associated with the large-scale purification of peptides have impeded the commercial exploitation of these products.^16^

To date, research in this field has primarily focused on peptides’ antioxidant and blood-pressure-lowering effects.^2,3^ Peptides can act as antioxidants in many ways, including by inactivating reactive oxygen species, scavenging free radicals, chelating pro-oxidant transition metals, reducing hydroperoxides, and inhibiting linoleic acid oxidation.^16^ Peptides effective in preventing or treating hypertension mainly work by inhibiting angiotensin-converting enzyme (ACE) activity; ACE is an integral part of blood pressure regulation and electrolyte homeostasis.^17^

In this work, hemp (*Cannabis sativa* L.) bran (HPB), a byproduct of the hemp seed food-processing chain, was assessed as a substrate for the preparation of bioactive compounds possessing antioxidant and antihypertensive properties. Several research articles have been previously published on the bioactivity of protein hydrolysates, fractions, or isolated peptides from hemp seed and meal.^7−10,18−23^ However, the specific HPB substrate, the coarser fraction of hemp meal (the part that remains after grinding and sieving the meal to obtain the flour, representing about 10% of the seed), has been only marginally studied to date.^24,25^ As reported by Pojić et al.,^24^ HPB contains proteins whose amount (about 10–20%, w/w) varies depending on the sieving mesh size. Different biotransformation processes (enzymatic hydrolysis, microbial fermentation, and solid-state fermentation by the oyster mushroom *Pleurotus ostreatus*) have recently proved that HPB can yield products with antioxidant and antihypertensive properties.^25^ The antihypertensive activity was particularly impressive: the IC_50_ values for ACE inhibition of bioprocessed samples were similar to those in protein hydrolysates from the whole hemp seed.^25^ To identify the peptides responsible for the bioactivity detected in bioprocessed HPB, the byproduct was hydrolyzed by Alcalase and divided by membrane ultrafiltration into four fractions of different molecular weights (MWs). The ability of protein-derived bioactive peptides to exert their activity is strictly dependent on their structure. To better understand the relationship between the peptides’ structural features and their bioactivity, peptides in the low-MW fraction were identified by high-resolution mass spectrometry coupled with sequence screening in the BIOPEP and BioPep DB databases and subsequently analyzed. The results of this work may be useful for promoting the recovery of HPB byproducts to develop value-added products that can be used by the food/pharmaceutical industries.

## Materials and Methods

### Raw Material and Chemicals

Milled hemp bran (HPB, the byproduct that remains when hemp seeds are mechanically pressed, ground, and sieved with a mesh size of 350 μm) was obtained from Hemp Positive World, Cesena, Italy. The analytical-grade reagents were made by Merck (Darmstadt, Germany). The precast gels, bovine serum albumin (BSA), MW marker for sodium dodecyl sulfate-polyacrylamide gel electrophoresis (SDS-PAGE), and mass spectrometry solvents were all obtained from Bio-Rad (Hercules, CA, USA).

### Protein Extraction

The procedure described by Setti et al.^25^ was used to extract the protein from HPB. Briefly the procedure consists of mixing the HPB with distilled water in a ratio of 1:20 (w/v) and adjusting the pH to 10.0 (by adding 2 N NaOH). The mixture was stirred for an hour and then centrifuged (8000*g*, 30 min, 4 °C). The supernatant was precipitated isoelectrically by adding 2 N HCl to achieve a pH of 5.0. The mixture was centrifuged (8000*g*, 10 min, 4 °C), and the resulting protein pellet was suspended in deionized water and further homogenized. This suspension was then adjusted to pH 7 using 2 N NaOH and freeze-dried in a Heto Power Dry LL300 freeze dryer (Thermo Fisher Corp.). The yield of hemp bran protein extract (HPBPE) was calculated as the ratio (%, w/w) of the dry mass of HPBPE to the amount of the original raw material.

### Enzymatic Hydrolysis

The HPBPE was first dissolved in deionized water (12.5%, w/v) and hydrolyzed with Alcalase 2% (v/v) at pH 8 for 2 h at 50 °C, as previously reported for the same substrate.^25^ The enzyme was inactivated, stopping the reaction, by heating the HPBPE to 85 °C for 15 min. After cooling to room temperature, the solution was centrifuged (14,000*g*, 20 min, 4 °C). The supernatant, Alcalase hydrolysate, was immediately collected and stored at −80 °C. The enzymatic digestion was carried out in triplicate; the three samples were combined (hereafter referred to as Alc) for fractionation, SDS-PAGE analysis, and bioactivity assays. The yield of hydrolyzed protein was calculated as the ratio (%, w/w) of the dry mass of Alc to the dry mass of HPBPE.

### Fractionation of Protein Hydrolysate by Membrane Ultrafiltration

The Alc sample was fractioned by membrane ultrafiltration on a 50 mL Amicon stirred cell (Millipore, USA). Three regenerated cellulose membranes (Millipore, USA) with cutoffs of 5, 3, and 1 kDa were used in sequence, creating four fractions: F1 with MW >5 kDa, F2 with MW between 5 and 3 kDa, F3 with MW between 3 and 1 kDa, and F4 with MW <1 kDa. The fractions were freeze-dried in a Heto Power Dry LL300 freeze dryer (Thermo Fisher Corp.) and then stored at −20 °C. Each fraction’s yield was assessed as the ratio (%, w/w) of the dry mass of the fraction to the dry mass of HPBPE.

### Protein Pattern Analysis (Tricine SDS-PAGE)

The protein pattern of each sample was analyzed on hand-cast 4% stacking, 10% separating, and 16% (w/v) separating tricine SDS-PAGE with the Mini-PROTEAN equipment (Bio-Rad, Hercules, CA, USA). Bio-Rad also provided the Precision Plus Protein Standard, which was used to determine the MW of each sample.

### Evaluation of Protein Content (Kjeldahl Assay)

The Kjeldahl method was used to determine the protein content. Briefly, 1.0 g (d.w.) of the sample was mixed with 10 mL of a 95:5 (v/v) H_2_SO_4_/H_3_PO_4_ solution and incubated at 420 °C for 180 min. Distillation was performed by adding 32% (v/v) NaOH; the sample was then titrated using 0.1 N H_2_SO_4_. Results were expressed in g proteins/100 g sample, applying the protein conversion factor *N* = 6.25.

### Evaluation of Degree of Hydrolysis (DH)

The DH of HPBPE hydrolysates was described as the quantity of soluble protein in 10% (w/v) trichloroacetic acid (TCA) compared to the total protein content of the sample (in percent).^26^ A 5 mL aliquot of 2 h Alcalase digested sample was combined with 5 mL of 20% (w/v) TCA. After the mixture stood for 5 min at room temperature, it was centrifuged (14,000*g*, 10 min, 4 °C), and the supernatant was analyzed by the Kjeldahl assay to obtain the Alc 10% TCA-soluble nitrogen. This value was used in the following equation to calculate the DH (%):
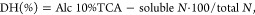
where total *N* is the total nitrogen content in undigested HPBPE (g) measured by the Kjeldahl assay.

### Assays of Antioxidant Activity

Antioxidant activity was ascertained by the following assays: ABTS (2,20-azino-bis-3-ethylbenzothiazoline-6-sulfonic acid) and DPPH (1,1-diphenyl-2-picrylhydrazyl) radical scavenging activity (ARSA and DRSA, respectively), ferrous ion-chelating ability (FCA), and ferric-reducing antioxidant power (FRAP). Lyophilized samples were dissolved in water at 10% (w/v) concentration and centrifuged (14,000*g*, 10 min); the supernatant was collected and adjusted to pH 7. All analyses were performed in triplicate on a microplate scale (SPARK 10M, TECAN, Switzerland). The results were given as IC_50_ values (mg of lyophilized sample/mL with 50% activity).

The ARSA was determined by following the procedure reported by Setti et al.^25^ The ABTS stock solution (7 mM in 2.45 mM K_2_S_2_O_8_) was diluted using sodium acetate (20 mM, pH 4.5) until an absorbance of 0.70 ± 0.02 at 734 nm was achieved. Then, 198 μL of this solution mixed with 2 μL of the sample was incubated in the dark at room temperature for 30 min. The absorbance was measured at 734 nm. A blank of distilled water was used (instead of the sample), and ascorbic acid (AA) served as a positive control.

The DRSA was also determined using the method of Setti et al.^25^ A 20 μL aliquot of the sample was mixed with 180 μL of 50 μM DPPH solution in methanol. After the mixture was incubated in the dark at room temperature for 30 min, the absorbance was measured at 517 nm. The blank and the positive control were the same as those for determining the ARSA.

The ferrous ion-chelating activity was also measured according to the method of Setti et al.,^25^ with slight modifications. The sample (25 μL) was mixed with 100 μL of 300 μM ferrozine and 100 μL of 50 μM FeSO_4_ and incubated at room temperature for 10 min. The absorbance was then measured at 562 nm. The positive control consisted of ethylenediaminetetraacetic acid (EDTA).

The FRAP was measured using a method based on the fact that the Fe^3+^-TPTZ (2,4,6-tripyridyl-*s*-triazine) complex reduces to Fe^2+^-TPTZ at a low pH when antioxidants are present.^27^ The FRAP reagent was prepared by mixing 1 mL of a 10 mM TPTZ solution in 40 mM HCl, 1 mL of 20 mM FeCl_3_, and 10 mL of 300 mM acetate buffer with pH 3.6. Aliquots of the sample (10 μL) were blended with 300 μL of the FRAP reagent; after incubation at room temperature for 10 min, the absorbance of the mixture at 593 nm was measured. AA was used as the positive control.

### Assay of the ACE-Inhibitory Activity

The ACE-inhibitory activity was measured using the method of Sentandreu and Toldra.^28^ Aliquots of the sample (50 μL) were mixed with the ACE solution (50 μL, 15 mU/mL) and preincubated at 37 °C for 10 min. The addition of 200 μL of preheated (37 °C) 0.45 mM *o*-aminobenzoylglycyl-*P*-nitro-l-phenylalanyl-l-proline (Abz-Gly-Phe(NO_2_)-Pro), dissolved in 150 mM Tris buffer (pH 8.3) containing 1.125 M NaCl, started the reaction. The mixture was incubated at 37 °C for 30 min. The fluorescence generated by the release of the *o*-aminobenzoylglycine (Abz-Gly) group was measured using excitation and emission wavelengths of 355 and 405 nm, respectively, using a fluorometer microplate reader (SPARK 10M, TECAN, Switzerland). The standard curve was obtained by using different concentrations of Abz-Gly (from 5 to 30 μM). The IC_50_ values (mg/L) were determined by nonlinear regression analysis. The spectrophotometric data were fit to the log (inhibitor) vs the response model, which was generated by GraphPad Prism 8.0 (GraphPad Software, San Diego, CA, USA).

### Peptide Identification Using High-Resolution Mass Spectrometry

The peptidomic profile of fraction F4 (<1 kDa) was analyzed by UHPLC (UHPLC Ultimate 3000, Thermo Scientific, San Jose, CA, USA) coupled with high-resolution mass spectrometry (Q Exactive Hybrid Quadrupole-Orbitrap Mass Spectrometer, Thermo Scientific, San Jose, CA, USA). A Zorbax C18 column was used for the chromatographic separation (Zorbax SB-C18 Reversed-phase, 2.1 × 50 mm, 1.8 μm particle size, Agilent Technologies, Santa Clara, CA, USA). Further information about the mobile phases, the elution gradient, and the mass spectrometry parameters is provided by Martini et al.^29^*De novo* peptide sequencing was carried out with the Pepnovo software (http://proteomics.ucsd.edu/ProteoSAFe/) with the following parameters: fragment mass tolerance, ±0.12 Da; peptide mass tolerance, ±5 ppm; enzyme, none; and oxidation (M) and phosphorylation (ST) as variable modifications. Each identified peptide was confirmed by manual inspection of the MS and MS^2^ spectra. Two different databases (BIOPEP and BioPep DB) were used for the identification of peptides with previously reported biological activity.^30,31^

### Statistical Analysis

Each analysis was performed in triplicate, and results are reported as mean value ± SD. Statistical testing was performed using the SPSS software (SPSS16, Inc., USA). Determining whether differences among means were significant was accomplished by the use of a one-way analysis of variance (ANOVA) with Tukey test and a significant level of *p* < 0.05.

## Results and Discussion

**Figure 1:**
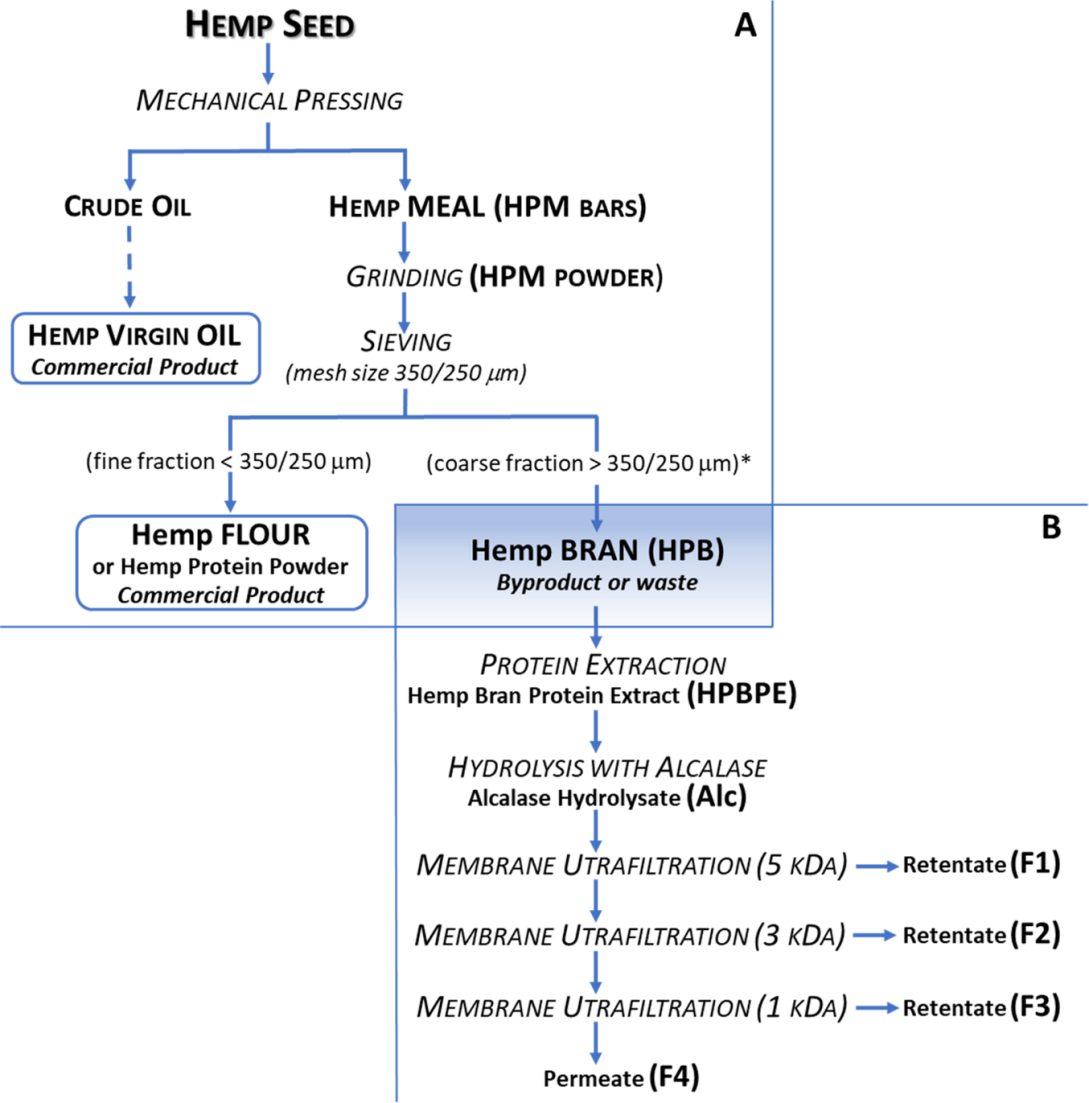
Simplified flow diagram of the (A) industrial processing of hemp seed for the production of the two commercial products, virgin oil and flour, and (B) experimental processing of the hemp bran (HPB) byproduct to produce the samples analyzed in the present work. Abbreviations in parentheses: Alc, HPBPE hydrolyzed by Alcalase; F1, Alc peptide fraction >5 kDa; F2, Alc peptide fraction from 3 to 5 kDa; F3, Alc peptide fraction from 1 to 3 kDa; F4, Alc peptide fraction <1 kDa; HPBPE, HPB protein extract; HPM, hemp meal. *The HPB used in the present work was produced by the company using a 350 μm mesh size.

Experimental samples were obtained from a byproduct of the hemp seed food-processing chain (Figure 1). The seeds are processed by the food industry to provide two main commercial products: virgin oil and flour (the latter also known as hemp protein powder) (Figure 1A). The first step of the process is the mechanical pressing of the seeds, which yields crude oil and small cylindrical bars of hemp meal (HPM). The oil is subsequently refined into virgin oil. The bars are ground into powder and sieved, which separates the flour from the larger particles (larger than 250–350 μm, depending on the degree of flour refining). The coarser fraction (hemp bran, HPB), representing about 10% of the seed, is normally used for animal feed or even discarded. This fraction is relatively high in proteins, although the percentage depends on the seed varieties and extraction procedures. Pojić et al.^24^ estimated that HPB preparations including particles larger than 250 and 350 μm contained approximately 20 and 11% (w/w) protein, respectively. In the present work, HPB > 350 μm was used; the protein content, as previously reported by the authors, was about 20% (w/w).^25^ The procedure to convert HPB into added-value hydrolysates is reported in Figure 1B. The first two steps (chemical extraction and hydrolysis with Alcalase) reproduced a process previously used by the authors to generate, from the HPB matrix, hydrolysates with improved bioactive properties.^25^ This procedure was further extended in the present work by adding sequential membrane ultrafiltration steps to separate the hydrolysates into four fractions: MW >5 kDa (F1), 5–3 kDa (F2), 3–1 kDa (F3), and <1 kDa (F4). Some papers reported the increased bioactivity of HPM protein hydrolysates after peptide separation in different fractions, according to their MW or hydrophobicity.^8−10^ Many other examples exist in the literature for the fractionation of protein hydrolysates from other matrices.^12,14^ The addition of a purification step (such as ultrafiltration or chromatographic separation) raises the processing costs; however, on an industrial scale, the added cost may be justified by the greater commercial value of the product due to the increased bioactivity. Further advantages in the fractionation of hydrolysates could derive from the increased resistance to physiological digestion and the higher bioavailability of low MW peptides with respect to mixtures of more complex MW distribution.

### Protein Profile, DH and Yield of Hydrolysate, and Ultrafiltration Fractions

**Figure 2:**
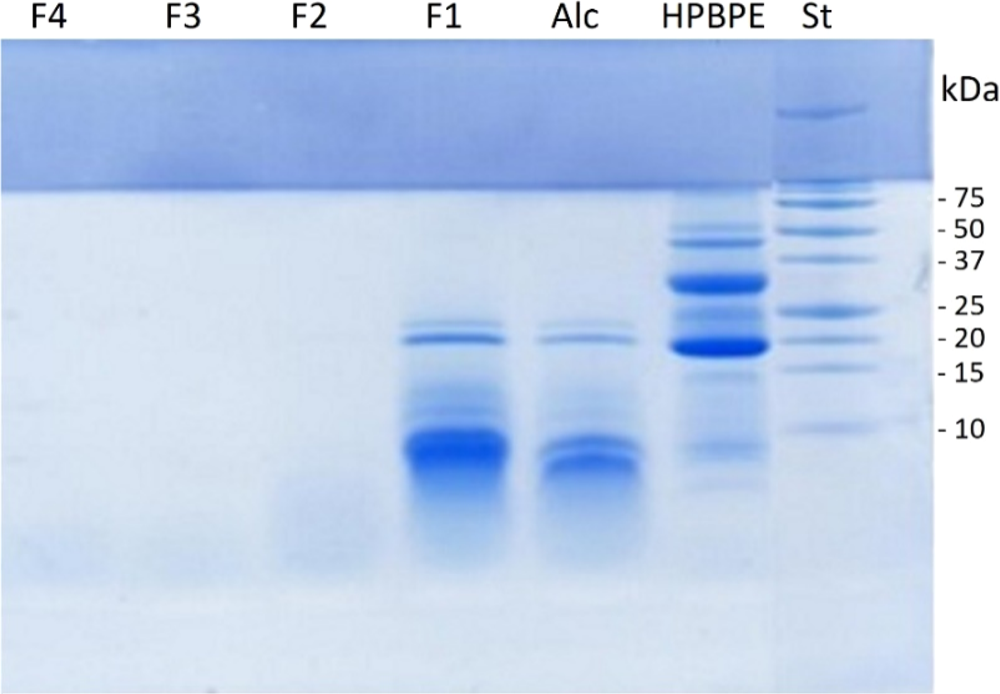
Tricine SDS-PAGE of the hemp bran protein extract (HPBPE); its hydrolysate with Alcalase (Alc); and peptide fractions F1 (>5 kDa), F2 (3–5 kDa), F3 (1–3 kDa), and F4 (<1 KDa). St: MW marker.

The efficiency of the Alcalase digestion of HPBPE and of hydrolysate fractionation by membrane ultrafiltration was followed by checking the protein profile on tricine SDS-PAGE (Figure 2). The initial protein extract (HPBPE) showed bands with MW up to 75 kDa. After hydrolysis, bands with MW higher than 20 kDa disappeared, leaving smaller bands, partially unresolved, that confirmed the efficacy of the proteolytic reaction. Fractionation was able to separate peptides with MW >5 kDa in the F1 sample, which formed clearly visible smeared bands on SDS-PAGE, the most intense with a MW close to 10 kDa. These bands totally disappeared in F2 to F4 samples, which contained peptides with progressively lower MW, as expected for the membrane cutoff of ultrafiltration steps. The DH (indicating the percent of bonds available for proteolytic hydrolysis that were actually cleaved) is a standard parameter for monitoring the level of proteolysis. The DH of HPBPE by Alcalase was 27.46% (Table 1), which falls within the range of previously reported single enzymatic treatments of HPM protein extracts (reported as 19.7 to 47.5%^22^ and as 39.1%^9^). The yield of the HPBPE hydrolysate and of each fraction obtained by membrane ultrafiltration is also reported in Table 1. This parameter is another important yardstick of the efficiency of enzymatic hydrolysis (a higher yield of hydrolysate/peptide fractions indicates an increase in protein breakdown); it is also relevant for determining the products’ potential for commercial exploitation in the food industry (higher yield potentially meaning higher economic viability). The enzymatic hydrolysis of HPBPE with Alcalase caused 70% of the peptide yield, again in the range of previous reports for HPM hydrolysates (reported as 16 to 43%^22^ and as 65.7%^9^). The separation of the protein hydrolysate (Alc) by membrane ultrafiltration indicated that the lowest MW fraction (F4) was present in a higher proportion (35.83%), followed by F1 (21.13%), when compared to fractions F2 and F3 (5.26 and 4.84%, respectively). A similar trend was obtained for HPM protein extract ultrafiltration fractions by Girgih et al.^9^

**Table 1:** Percent Yield of Hemp Bran Protein Extract (HPBPE); Its Hydrolysate with Alcalase (Alc); and the Alc Peptide Fractions F1 (>5 kDa), F2 (3–5 kDa), F3 (1–3 kDa), and F4 (<1 kDa)^a^

sample	yield (%)	DH (%)
HPBPE	7.05 ± 0.57	NA
Alc	70.02 ± 1.79	27.46 ± 0.41
F1	21.13	NA
F2	5.26	NA
F3	4.84	NA
F4	35.83	NA

a The degree of hydrolysis (DH, %) was measured for the HPBPE hydrolysate with Alcalase (Alc). NA means not applicable.

### Antioxidant Properties

**Figure 3:**
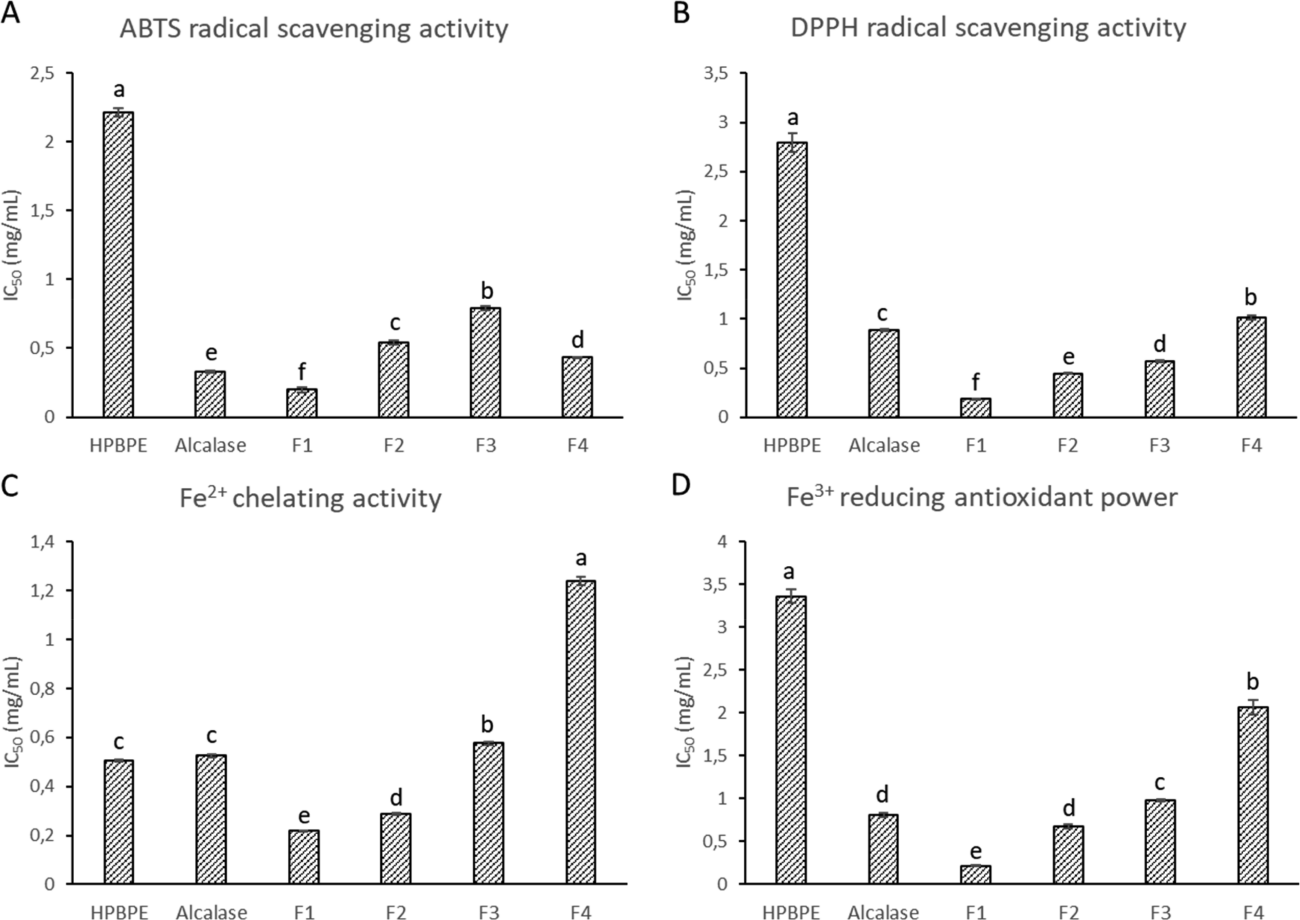
Antioxidant activity, expressed as IC_50_ values (mg/mL), of the hemp bran protein extract (HPBPE); its hydrolysate with Alcalase (Alc); and peptide fractions F1 (>5 kDa), F2 (3–5 kDa), F3 (1–3 kDa), and F4 (<1 KDa). (A) ABTS radical scavenging activity; (B) DPPH radical scavenging activity; (C) ferrous ion-chelating activity; and (D) ferric-reducing antioxidant power. Means followed by the same letter did not differ significantly (Tukey test, *p* > 0.05).

The antioxidant properties of HPBPE, Alc, and the fractions F1 to F4 were evaluated by four different assays: ARSA, DRSA, FCA, and FRAP. The ARSA and DRSA measure the ability to scavenge stable radical species (ABTS^·^ and DPPH^·^, respectively). The FCA assay evaluates the total antioxidant capacity indirectly, measuring the Fe^2+^ chelation by ferrozine to form a stable complex. The FRAP assay evaluates the ability of antioxidants to directly reduce Fe^3+^ as an electron-donating activity indicator. The results of all assays are reported as IC_50_ values; the lowest value indicates the highest activity (Figure 3).

The results of the ARSA, DRSA, and FRAP assays had similar trends (Figure 3A,B,D). Enzymatic hydrolysis with Alcalase strongly increased the antioxidant activity, compared to HPBPE, as exerted by the three mechanisms. Indeed, the IC_50_ values decreased with respect to those of the undigested sample, of about 85, 69, and 76%, respectively. The fourth assay, FCA, showed a different trend, as hydrolysis with Alcalase did not have a positive effect on Fe^2+^ chelation with respect to HPBPE (Figure 2C). The IC_50_ values of the two samples were indeed comparable (0.50 and 0.53 mg/mL for undigested and digested samples, respectively). Similar results for ARSA and FRAP assays were previously reported for the HPB Alcalase hydrolysate.^27^ Furthermore, enzymatic hydrolysis (by Alcalase or other enzymes) increased the antioxidant properties of proteins extracted from HPM.^7,8,10,18−20,32^ The hydrolysis of protein, as already stated for many other substrates, allows the release of specific protein fragments that, although inactive within the parent proteins, become active when free in the solution. The fractionation of the Alc hydrolysate by membrane ultrafiltration increased the antioxidant activities of all F1 samples that had IC_50_ values (0.19, 0.18, 0.22, and 0.21 mg/mL for ARSA, DRSA, FCA, and FRAP, respectively) significantly lower than those of the unfractionated sample (0.33, 0.88, 0.53, and 0.81 mg/mL for ARSA, DRSA, FCA, and FRAP, respectively). The subsequent fractions, containing peptides with decreasing sizes, displayed a lower antioxidant activity than F1. Fractions F2, F3, and F4 had progressively increasing ARSA, DRSA, FCA, and FRAP IC_50_ values (with the exception of the ARSA value of sample F4, which was lower than that of the F3 and F2 samples). No data are available in the literature for peptide fractions from HPB protein extracts. The ultrafiltration fractions from HPM had antioxidant activity values that were dependent on the MW.^8^ An opposite trend was observed for HPB fractions for DRSA (smaller fractions showing higher potency), but the trend was similar for FCA and FRAP. Tang et al.^7^ demonstrated that increasing the time of HPM protein extract hydrolysis by different enzymes from 2 to 4 h (thus increasing the percentage of smaller peptides in solution) led in some cases to decreased DRSA, FCA, and FRAP values. Das^20^ also reported an inverse correlation between the degree of hydrolysis and the radical scavenging activity for the HPM protein extract hydrolyzed with different enzymes. Other examples of increasing antioxidant activity in higher MW fractions of protein hydrolysates come from other substrates. He et al.^11^ observed that high-MW (5–10 kDa) fractions of the rapeseed protein hydrolysate exhibited the strongest chelating capacity. High-MW fractions of the pigeon pea protein hydrolyzed by pancreatin showed the highest DRSA and FRAP values.^13^ A decrease in DRSA activity with a decrease in the peptide sizes was observed for the yellow stripe trevally protein hydrolysate prepared using Alcalase.^6^

During hydrolysis, a mixture of small peptides and free amino acids is formed. Their antioxidative potential depends on the size of the generated peptides and the nature and sequence of their amino acid residues. In these mixtures, low-MW peptides are generally the most active for specific factors (easier steric interaction with the substrate, higher hydrophilicity and solubility, exposure of hydrophobic side chains, etc.) and their combination. The decrease of antioxidant activity with decreasing MW, as observed for F2 to F4 samples, could be related to the soluble peptide aggregates that form in the hydrolysates, hiding hydrophobic amino acid side chains and reducing reactive peptides. Alternatively, the decrease could be due to the formation of free amino acid residues (which exhibit lower antioxidant properties than amino acid sequences). Although the molecular basis of our findings is not yet known with certainty, the results of the antioxidant activity assays are promising. They confirmed the evidence of a preliminary work indicating that proteins extracted from this coarsest part of HPM (and not just from the entire HPM, as already established by other authors) have antioxidant properties, which can be significantly increased by Alcalase digestion.^25^ Besides, the addition of a purification step by membrane ultrafiltration with a cutoff of 5 kDa (to isolate peptides with MW higher than 5 kDa) can further increase this activity because the combined effects of several properties, including peptides’ ability to scavenge free radicals, act as chelating agents of metal ions, and donate hydrogen.

### ACE-Inhibitory Activity

ACE catalyzes two important reactions that are responsible for constricting blood vessels, which in turn leads to elevated blood pressures.^9^ An *in vitro* ACE-inhibitory activity assay was used to screen the antihypertensive potential of HPBPE, Alc, and the fractions F1 to F4. Results are expressed as IC_50_ values (mg/L), indicating the concentration of the sample able to induce a 50% inhibition of the enzyme (Table 2). The hydrolysis of HPBPE by Alcalase had a strong positive effect on the ACE-inhibitory activity as evidenced by an IC_50_ value of 80 mg/L. Accordingly, these findings reveal that ACE-inhibitory peptides were released from HPB proteins during enzymatic hydrolysis. The IC_50_ value of the Alc sample was in the range of previous results obtained for HPB proteins hydrolyzed by Alcalase (176 mg/L^25^) and for HPM proteins hydrolyzed by different enzymes (16–228 mg/L^21^). Similar IC_50_ values were observed for protein hydrolysates from other substrates (140 mg/L for green soybean Alcalase hydrolysate^33^ and 224 and 226 mg/L for soybean and lupin pepsin digests, respectively^34^). Fractionation based on peptide MW was not as efficient as enzymatic hydrolysis to increase the inhibitory activity of the Alc sample. The ACE-inhibitory activity of ultrafiltered samples F3 and F4 was slightly increased with respect to the Alc sample (72 mg/L, both), but the F1 and F2 fractions had higher or equal values (108 and 83 mg/L, respectively). Membrane ultrafiltration of the HPM protein hydrolysate, reproducing the effects of gastrointestinal digestion, produced peptide fractions that were less inhibitory toward ACE than the original hydrolysate. The IC_50_ values were higher in the low-MW fractions (1050 and 1170 mg/L for <1 and 1–3 kDa, respectively) than the unfractionated sample (670 mg/L).^9^ Conversely, reports on cod frame protein hydrolysate,^5^ shrimp,^4^ and sesame protein^15^ indicate that the ACE-inhibitory activity increases as the MW of peptide fractions (produced by ultrafiltration) decreases, as observed in the present study. This variability can be explained by the fact that size is not the only parameter affecting the peptides’ ACE-inhibitory activity; the activity is also dependent on the presence of hydrophobic residues (aromatic or branched-chain) and on their position in the amino acidic sequence.^17,35^ In addition, in complex mixtures, the synergistic effect of different peptides can also be a factor, increasing the ACE-inhibitory potency of protein hydrolysates.^9^ Independent of the mode of ACE inhibition, the IC_50_ values of the HPB hydrolysate and of the single fractions appear to be significant. They are comparable with values measured for other-sources protein hydrolysates, as previously reported, but also for pure peptides isolated from HPM hydrolysates^19^ and reference antihypertensive peptides.^28^ These data suggest that both the hydrolysate and the single fractions can be considered as a valuable source of ACE-inhibitory peptides, with the promise of application in the formulation of functional foods or dietary supplements that could prevent or treat hypertension.

**Table 2:** ACE-Inhibitory Activity of the Hemp Bran Protein Extract (HPBPE); Its Hydrolysate with Alcalase (Alc); and Alc Peptide Fractions F1 (>5 kDa), F2 (3–5 kDa), F3 (1–3 kDa), and F4 (<1 kDa) (Means Followed by the Same Letter Did Not Differ Significantly (Tukey Test, *P* > 0.05))

sample	ACE-inhibitory activity (IC_50_ mg/L)^a^
HPBPE	2798 ± 0.121^a^
Alc	83 ± 0.034^bB^
F1	108 ± 0.001^bA^
F2	83 ± 0.004^bB^
F3	72 ± 0.002^bC^
F4	72 ± 0.001^bC^

a Statistical analysis was performed considering the samples all together and without HPBPE (lowercase and uppercase letters, respectively).

### Peptidomic Profile of Alcalase Hydrolysate Fraction F4 (<1 kDa)

With the aim to identify alleged bioactive peptides, fraction F4 was subjected to high-resolution mass spectrometry analysis. The F4 fraction was selected for peptidomic investigation for its high biological potential (ACE-inhibitory and antioxidant activities) and for the presence of short peptide sequences, which are actually less susceptible to the action of gastrointestinal and brush-border proteases and, thus, potentially bioavailable.^36^ The full list of identified peptides as well as the mass spectrometry data is provided in Table S1. A total of 239 peptides having 2 to 9 amino acid residues and 4 free amino acids were detected in fraction F4. Among the identified peptides, 39 were dipeptides, 78 were tripeptides, 37 were tetrapeptides, and 85 contained 5 or more residues.

Some of the identified peptides have already been characterized as antioxidant or ACE-inhibitory peptides. Table 3 bears the identified peptides and amino acids formerly described as antioxidant compounds. The C-terminal bulky Y-residue recorded in three dipeptides (AY, VY, and TY) and one tripeptide (LLY) is pivotal in determining the antioxidant activity of these peptides. The tripeptide LLY has already proven to be a strong antioxidant peptide both *in vitro* in cell culture and *in vivo* in mice under oxidative stress conditions.^37^ Previously, it was demonstrated that the occurrence of an antioxidant amino acid (Y or W) at the C-terminus is of paramount importance for the ABTS and hydroxyl radical scavenging activities of bioactive peptides.^38^ Several di- and tripeptides containing Y at the C-terminus were found in the fraction F4 (Table S1), which can contribute to the antioxidant properties of this fraction.

**Table 3:** Peptides and Amino Acids with Previously Described Antioxidant Properties Identified in Fraction F4 (<1 kDa)

sequence	activity
LLY	ABTS radical scavenging
	peroxyl radical scavenging
LLR	ABTS radical scavenging
	inhibition of lipid peroxidation
IR	peroxyl radical scavenging
TY	ABTS radical scavenging
	inhibition of lipid peroxidation
	metal chelating ability
VY	ABTS radical scavenging
	inhibition of lipid peroxidation
LH	inhibition of lipid peroxidation
EL	DPPH radical scavenging
	hydroxyl radical scavenging
	superoxide anion scavenging activity
LK	peroxyl radical scavenging
AY	inhibition of lipid peroxidation
H	hydroxyl radical scavenging
	inhibition of lipid peroxidation
R	hydroxyl radical scavenging
	inhibition of lipid peroxidation
F	hydroxyl radical scavenging
	inhibition of lipid peroxidation

A total of 35 peptides previously described as ACE-inhibitors were identified in fraction F4 and reported in Table 4. The tripeptides IVY and LIY, formerly isolated from wheat germ hydrolysate and human plasma, respectively, showed IC_50_ values lower than 1 μmol/L and could be the primary contributors to fraction F4’s ACE-inhibitory activity.^39^ The dipeptide VY showed a low IC_50_ value against ACE and behaved as a multifunctional bioactive peptide with antioxidant as well as DPP-IV and DPP-III inhibitory activities.^40^ Moreover, VY was found to be bioavailable in humans and effective *in vivo*, inducing a significant decrease in blood pressure in both highly and mildly hypertensive subjects with no observed changes in normotensive ones.^41,42^ In addition, other identified peptides with low or very low IC_50_ values (IVY, LVY, and LLF) have shown antihypertensive activity *in vivo* (in spontaneously hypertensive rats).^35,43^ According to studies of the structure–activity relationship, the presence of aromatic or hydrophobic amino acid residues (such as W, Y, F, I, L, and P) at the C-terminus and/or the amino acids G, I, L, R, and V at the N-terminus is pivotal for ACE inhibition.^44^ In this context, some peptides identified in fraction F4 (specifically, ISY, IGF, VSF, VTF, and VGL) might potentially inhibit the ACE activity. For example, the tripeptide ISY meets the structural requirement for ACE inhibition and shares the motif SY of the typical acknowledged ACE-inhibitory peptides such as SY, PSY, AHSY, LLPSY, and AKYSY.^45,46^ Similarly, the tripeptides VSF and VTF are precursors of the ACE-inhibitory dipeptides SF and TF and share the C-terminal residues with other potent ACE-inhibitory peptides (such as FQPSF, EGGSF, LTF, and ITF).^47,48^ These peptides play a role in the ACE-inhibitory activity of F4 and can be further exploited for their potential inhibitory effect.

**Table 4:** Peptides with Previously Described Angiotensin-Converting Enzyme (ACE)-Inhibitory Activity Identified in Fraction F4 (<1 kDa)^a^

sequence	IC_50_^b^
IVY	0.5 μmol/L
LIY	0.8 μmol/L
IIY	1.1 μmol/L
EY	2.7 μmol/L
AI	3.4 μmol/L
LVY	5.8 μmol/L
VY	7.1 μmol/L
VK	13 μmol/L
LVQ	14 μmol/L
AY	19 μmol/L
LGI	29 μmol/L
NY	32 μmol/L
IKY	34 μmol/L
VVF	35 μmol/L
ITF	49 μmol/L
FQ	51 μmol/L
LLP	57 μmol/L
SY	66 μmol/L
LLF	80 μmol/L
IVQ	95 μmol/L
LEE	100 μmol/L
SF	130 μmol/L
IA	153 μmol/L
LR	158 μmol/L
GY	210 μmol/L
ILP	270 μmol/L
LA	310 μmol/L
LTF	330 μmol/L
IR	695 μmol/L
FR	920 μmol/L
GI	1200 μmol/L
GL	2500 μmol/L
IE	n. a.^c^
EI	n. a.
LQ	n. a.

a Peptides are listed based on their inhibitory potency.

b IC_50_ is defined as the concentration of peptides required to inhibit 50% of the enzymatic activity. The values are from the BIOPEP and MBPDB databases.^32,33^

c n.a. means IC_50_ value not available.

Additional peptides with other biological activities than antioxidant and ACE-inhibition were detected in fraction F4 and reported in Table S2. The asserted biological activities included dipeptidyl peptidase-IV (DPP-IV) and dipeptidyl peptidase-III (DPP-III) inhibitory activities, glucose uptake stimulating activity, renin inhibition, and immunostimulating activities. All of these identified bioactive peptides were short sequences of two or three amino acids. The majority of these peptides (37 identified peptides) showed a DPP-IV inhibitory activity. DPP-IV, a brush-border prolyl-dipeptidyl peptidase, is involved in the degradation of the insulinotropic hormones known as incretins. It is considered a molecular target for managing type 2 diabetes.^49^ It is known that DPP-IV inhibitory peptides are generally characterized by a hydrophobic feature.^49^ Accordingly, the presence of Y, L, I, V, T, G, and A in the sequences of the identified di- and tripeptides listed in Table S2 likely boosted their efficacy, making them the main potential candidates as DPP-IV inhibitors.

In conclusion, this study highlights the potential use of HPB hydrolysate and fractions as multifunctional ingredients for the development of new healthy foods or for the pharmaceutical industry. Most of the bioactive peptides identified are short sequences of a few amino acids, potentially resistant to the gastrointestinal conditions. Indeed, some of these peptides have confirmed bioavailability in humans or rats. However, to validate the results, *in vivo* investigations are required to help us better understand the physiological significance of the alleged bioactivity. In the future, the production of hydrolysates enriched in bioactive peptides from the HPB protein extract may pave the way for new economic opportunities for this byproduct.

## Supporting Information Available

List of the peptides identified in hemp bran Alcalase hydrolysate fraction F4 (<1 kDa) (Table S1) and list of peptides and amino acids with previously described biological activity identified in the hemp bran Alcalase hydrolysate F4 (<1 kDa) (Table S2) (PDF)

## Abbreviations

**ABTS**: 2,2-azino-bis (3-ethylbenzothiazoline-6-sulfonic acid)
**ACE**: angiotensin-converting enzyme
**Alc**: HPBPE hydrolysates with Alcalase
**ARSA**: ABTS radical scavenging activity assay
**CVDs**: cardiovascular diseases
**DPPH**: 2,2-diphenyl-1-picrylhydrazyl
**DRSA**: DPPH radical scavenging activity assay
**EDTA**: ethylenediaminetetraacetic
**FRAP**: ferric-reducing antioxidant power assay
**FCA**: ferrous ion chelating activity assay
**F1**: Alc fraction with MW >5 kDa
**F2**: Alc fraction with MW between 5 and 3 kDa
**F3**: Alc fraction with MW between 3 and 1 kDa
**F4**: Alc fraction with MW <1 kDa
**HPB**: hemp bran
**HPBPE**: hemp bran protein extract
**HPM**: hemp meal
**MW**: molecular weight
**SDS-PAGE**: sodium dodecyl sulfate-polyacrylamide gel electrophoresis
**TCA**: trichloroacetic acid
**TPTZ**: 2,4,6-tri(2-pyridyl)-*s*-triazine
