# Supplementary material for: Antioxidant and Angiotensin I-Converting Enzyme (ACE) Inhibitory Peptides Obtained from Alcalase Protein Hydrolysate Fractions of Hemp (Cannabis sativa L.) Bran
Source: J Agric Food Chem. 2021 Aug 6;69(32):9220–8. doi: 10.1021/acs.jafc.1c01487 (PMC8389806; doi:10.1021/acs.jafc.1c01487)
Supplement: Supplementary file 1 — jf1c01487_si_001.pdf [file jf1c01487_si_001.pdf]

Table S1. List of the peptides identified in fraction F4. Lx means that the peptide was found both with leucine (L) or isoleucine (I).

m/z observed	*m/z* calculated	Error (ppm)	Charge	Peptide
132.1019	132.1019	0.00	1	Lx
156.0770	156.0768	1.26	1	H
166.0863	166.0863	0.00	1	F
175.1191	175.1190	0.55	1	R
189.1233	189.1234	-0.53	1	GLx
203.1390	203.1390	0.00	1	LxA
203.1390	203.1390	0.00	1	ALx
217.1546	217.1547	-0.46	1	VV
219.1338	219.1339	-0.46	1	TV
219.1338	219.1339	-0.46	1	SLx
221.0953	221.0954	-0.45	1	AM
231.1703	231.1703	0.00	1	LxV
233.1495	233.1496	-0.43	1	LxT
233.1496	233.1496	0.00	1	TLx
237.0903	237.0904	-0.42	1	SM
239.1026	239.1026	0.00	1	GY
244.1186	244.1186	0.00	2	YAPH
246.1447	246.1448	-0.41	1	VQ
246.1813	246.1812	0.41	1	VK
247.1289	247.1288	0.40	1	DLx
253.1182	253.1183	-0.40	1	SF
253.1182	253.1183	-0.40	1	AY
260.1603	260.1605	-0.77	1	QLx
260.1604	260.1605	-0.38	1	LxQ
260.1969	260.1969	0.00	1	LxK
261.1444	261.1445	-0.38	1	LxE
261.1445	261.1445	0.00	1	ELx
269.1130	269.1132	-0.74	1	SY
269.1608	269.1608	0.00	1	LxH
280.1290	280.1292	-0.71	1	FN
281.1495	281.1496	-0.36	1	VY
283.1287	283.1288	-0.35	1	TY

284.1352	284.1353	-0.35	1	HGA
288.1916	288.1918	-0.73	1	GVLx
288.1916	288.1918	-0.73	1	VGLx
288.2028	288.2030	-0.69	1	LxR
290.1709	290.1710	-0.34	1	GTLx
294.1446	294.1448	-0.68	1	FQ
294.1810	294.1812	-0.68	1	FK
295.1288	295.1288	0.00	1	FE
296.1239	296.1241	-0.68	1	NY
302.2075	302.2074	0.33	1	LxGLx
303.1449	303.1452	-0.99	1	FH
304.1500	304.1503	-1.16	1	VGE
304.1865	304.1867	-0.66	1	LxAT
310.1394	310.1397	-0.97	1	QY
310.1395	310.1397	-0.91	1	SGF
310.1395	310.1397	-0.64	1	YQ
311.1234	311.1238	-1.29	1	EY
318.2023	318.2023	0.00	1	VSLx
320.1815	320.1816	-0.31	1	LxST
322.1872	322.1874	-0.62	1	FR
331.1975	331.1976	-0.30	1	QLxA
331.1976	331.1976	0.00	1	VVN
332.1813	332.1816	-0.90	1	VVD
332.2177	332.2180	-0.90	1	LxSLx
333.1765	333.1769	-0.97	1	AGVS
333.1766	333.1769	-0.90	1	VTN
336.1918	336.1918	0.00	1	LxGF
338.1710	338.1710	0.00	1	ATF
338.1821	338.1823	-0.59	1	YR
340.1977	340.1979	-0.59	1	HLxA
342.2388	342.2387	0.29	1	LxLxP
344.2542	344.2544	-0.58	1	LxLxV
345.1767	345.1769	-0.52	1	TPAG
345.2131	345.2132	-0.29	1	LxVN
345.2241	345.2245	-1.16	1	LxGR
345.2494	345.2496	-0.58	1	VVK

346.1970	346.1973	-0.87	1	VVE
346.1971	346.1973	-0.39	1	VDLx
347.1559	347.1561	-0.58	1	VND
347.1923	347.1925	-0.58	1	LxQS
347.1924	347.1925	-0.29	1	SQLx
348.1763	348.1765	-0.57	1	LxSE
351.2024	351.2027	-0.85	1	KGF
352.1867	352.1867	0.00	1	VSF
358.2697	358.2700	-0.84	1	LxLxLx
359.2286	359.2289	-0.84	1	LxVQ
359.2288	359.2289	-0.28	1	VLxQ
359.2288	359.2289	-0.28	1	LxLxN
359.2651	359.2653	-0.56	1	LxVK
360.1877	360.1805	-0.22	1	AAAQ
360.2127	360.2129	-0.56	1	LxDLx
361.1714	361.1718	-1.11	1	NLxD
361.2079	361.2082	-0.83	1	TLxQ
362.1920	362.1922	-0.55	1	TELx
364.1350	364.1351	-0.09	1	SEE
364.2229	364.2231	-0.55	1	VVF
366.2024	366.2023	0.27	1	VTF
370.1608	370.1609	-0.27	1	TSY
370.1764	370.1761	0.81	1	FGF
187.1316	373.2558	0.32	2	VVR
374.2033	374.2034	-0.27	1	QNLx
375.1872	375.1874	-0.53	1	LxDQ
376.1715	376.1714	0.27	1	LxDE
377.2216	377.2217	-0.27	1	KVM
379.2087	379.2088	-0.26	1	FGR
380.1816	380.1816	-0.07	1	DVF
380.2177	380.2180	-0.79	1	LxTF
382.1974	382.1973	0.26	1	LxSY
191.6261	382.2449	0.00	2	LxLxH
387.2713	387.2714	-0.26	1	VLxR
389.1851	389.1853	-0.41	1	AMAP
389.2028	389.2031	-0.77	1	LxQE

389.2030	389.2031	-0.26	1	ELxQ
390.1869	390.1871	-0.51	1	LxEE
392.2542	392.2544	-0.51	1	LxLxF
394.2335	394.2336	-0.41	1	LxVY
400.1867	400.1867	0.00	1	FSF
401.2870	401.2871	-0.25	1	LxLxR
402.2457	402.2459	-0.50	1	LxNR
408.2493	408.2493	0.00	1	LxLxY
409.2196	409.2194	0.49	1	FSR
410.1669	410.1670	-0.24	1	NNY
413.2021	413.2035	-3.39	3	TQFYQNPPRS
415.2548	415.2551	-0.64	1	VSPLx
416.1815	416.1816	-0.24	1	YSF
208.6344	416.2616	0.00	2	ALxGR
416.2617	416.2616	0.24	1	LxQR
208.6470	416.2795	-0.19	2	AVVK
209.6297	417.2449	0.19	2	RSR
417.2455	417.2456	-0.24	1	ELxR
422.2032	422.2034	-0.47	1	FQQ
422.2032	422.2034	-0.47	1	QFQ
212.1337	423.2602	0.00	2	LxKY
425.1665	425.1667	-0.47	1	ENY
429.2343	429.2271	-0.10	1	ALxPE
429.2707	429.2708	-0.19	1	LxLxSP
215.6548	430.3024	0.00	2	LxAVK
431.2039	431.2037	0.46	1	HFQ
434.2245	434.2245	0.00	1	TTVN
435.2085	435.2086	-0.23	1	LxDTS
218.1393	435.2714	-0.46	2	FLxR
444.2452	444.2453	-0.23	1	TVPQ
444.2814	444.2817	-0.46	2	KPLxS
222.6557	444.2968	0.24	2	RLxR
446.2602	446.2537	-1.55	1	GVGLxT
447.2084	447.2011	-0.30	1	LxGEE
450.2345	450.2347	-0.36	1	NGLxF
452.2251	452.2252	-0.22	1	YNR

453.2457	453.2456	0.22	1	HLxPS
459.2199	459.2198	0.22	1	SQPAG
461.2719	461.2645	0.12	1	KGAVS
471.2561	471.2562	-0.21	1	LxQPN
473.2354	473.2354	0.00	1	TPQQ
237.6371	474.2671	-0.42	2	DALxR
237.6553	474.3035	-0.42	2	SVLxR
475.2147	475.2147	0.00	1	VGGDQ
476.2350	476.2351	-0.21	1	GVETA
478.2298	478.2296	0.42	1	YAPQ
479.2613	479.2613	0.00	1	FGQK
479.2987	479.2904	2.12	1	PLxLxH
243.6554	486.2962	0.12	2	LxTPR
487.3241	487.3166	0.50	1	AGVLxK
249.6734	498.3326	-0.61	2	PLxLxR
500.3076	500.3006	-0.55	1	LxALxPS
500.3077	500.3006	-0.22	1	LxLxAPS
250.6815	500.3482	0.39	2	LxVLxR
502.2873	502.2798	0.32	1	LxAGLxE
516.2411	516.2413	-0.39	1	NTGPQ
516.2777	516.2704	0.22	1	QGQALx
518.2460	518.2457	0.58	1	LxEQE
259.6446	518.2821	-0.39	2	EELxK
519.2299	519.2297	0.39	1	LxEEE
524.2714	524.2715	-0.19	1	YTLxQ
527.3187	527.3115	-0.02	1	LxLxNAP
528.3394	528.3319	0.37	1	LxVLxPS
529.3708	529.3635	-0.08	1	KVAVLx
530.2357	530.2285	-0.06	1	WNPN
268.6631	536.3118	-0.41	2	VVYR
543.3502	543.3428	0.18	1	LxLxGAVA
544.3091	544.3017	0.38	1	AGGQLxV
546.3136	546.3061	0.40	1	LxLxDVS
275.6710	550.3275	-0.08	2	YVLxR
551.3558	551.3479	1.20	1	PLxPKP
555.1931	555.1933	-0.36	1	YEDE

559.3085	559.3013	-0.12	1	PVGSLxS
563.2305	563.2307	-0.36	1	AAADSE
283.6320	566.2496	-0.27	2	FDER
569.3655	569.3584	-0.30	1	NLxPLxLx
571.3086	571.3013	0.05	1	DLxVPAG
286.1816	571.3489	-0.53	2	VSPLxR
577.2464	577.2464	0.00	1	VNDTE
291.1373	581.2678	-0.69	2	FDSGR
588.2625	588.2551	0.12	1	VGSPDN
295.1505	589.2867	-0.38	2	SSNKGP
295.1504	589.2940	-0.68	2	DGELxR
598.3208	598.3122	2.24	1	LxEHVT
301.1687	601.3231	-0.39	2	DLxTPR
605.2713	605.2712	0.17	1	DMSPR
609.2883	609.2806	0.66	1	AYVADA
617.2676	617.2678	-0.28	1	SWNPN
312.1460	623.2785	-1.65	2	LxFGSCP
623.3511	623.3439	0.01	1	HLxGLxSP
624.2733	624.2736	-0.48	1	RGDYN
315.2026	629.3908	-0.14	2	LxLxAGQK
631.3043	631.2973	-0.48	1	ADPSNK
316.1795	631.3522	-0.63	2	SGARLxQ
634.3193	634.3122	-0.28	1	APGQVY
640.4389	640.4319	-0.42	1	PLxLGLxK
643.3658	643.3588	-0.51	1	VEVPSE
322.6900	644.3653	0.26	2	AERGVLx
645.3454	645.3381	0.03	1	LxDGDLxL
323.6871	646.3598	-0.27	2	LxFGGPR
329.6978	658.3810	0.09	2	AGLxLxER
660.3566	660.3490	0.43	1	VLxVDSQ
662.2993	662.2992	0.15	1	QVEEAS
331.6680	662.3217	-0.51	2	CAELxRA
664.3775	664.3704	-0.26	1	QPGLxLxH
334.1854	667.3562	0.20	2	HRVQGA
336.2133	671.4199	-0.89	2	LxGGRLxGV
337.1972	673.3806	-0.99	2	EGNLxLxK

673.3875	673.3806	-0.57	1	ADNLxLxK
341.6612	682.3082	-0.68	2	SGFDRT
342.6691	684.3239	-0.23	2	LxDVNSH
685.3880	685.3807	0.11	1	GAGVVPVS
686.3718	686.3646	-0.15	1	LxSPADLA
350.2053	699.3963	-0.20	2	LxDVKPGA
702.3666	702.3595	-0.31	1	LxDAAALE
712.4239	712.4167	-0.06	1	LxELxVPAA
357.7109	714.4072	-0.01	2	LxDLxTPR
721.4087	721.4090	-0.42	1	VSSSTLxK
361.6768	722.3395	-0.52	2	FDGEVR
364.7062	728.3977	0.11	2	PSQAGLxR
732.3154	732.3159	-0.68	1	EGSGATNP
370.1979	739.3813	-0.17	2	GRFVNF
755.4293	755.4225	-0.64	1	LxTVASPPA
378.6797	756.3450	-0.12	2	PEEEPR
760.3541	760.3473	-0.69	1	PDDVVLxC
381.2004	760.3868	-0.69	2	DLxFNPR
783.4606	783.4538	-0.61	1	LxDPALxAALx
783.4607	783.4611	-0.38	1	LxVDGLxVPA
396.2019	791.3861	4.06	2	LxAWSLxSD
802.4301	802.4232	-0.49	1	NLxTLxLxGDG
402.7094	804.4025	2.10	2	LxVSGNDVT
404.2217	807.4286	0.20	2	ELxGGRLxY
411.1809	821.3523	2.68	2	QEEDLxST
278.1676	832.4814	-0.48	3	SETVLxKR
418.2191	835.4308	0.00	2	VFDGELxR
444.7121	888.4097	-0.04	2	VVDNDGNR
890.4358	890.4294	-0.94	1	PGGGNLxQFT
447.7378	894.4607	0.53	2	SAERGVLxY
521.7745	1042.5389	-4.26	2	LxTSLxRNAHM

Table S2. Peptides and amino acids with previously described biological activity identified in the fraction F4 (<1 kDa).

**Sequence**	**Biological activity**
H	Antioxidant
F	Antioxidant
R	Antioxidant
GL	ACE inhibitor, DPP-IV inhibitor
GI	ACE inhibitor, DPP-IV inhibitor
LA	ACE inhibitor, DPP-IV inhibitor, DPP-III inhibitor
IA	ACE inhibitor, DPP-IV inhibitor
AL	DPP-IV inhibitor
AI	ACE inhibitor
VV	DPP-IV inhibitor
TV	DPP-IV inhibitor
SL	DPP-IV inhibitor
SI	DPP-IV inhibitor
LV	DPP-IV inhibitor<br>Glucose uptake stimulating peptide
IV	Glucose uptake stimulating peptide
LT	DPP-IV inhibitor
TL	DPP-IV inhibitor
TI	DPP-IV inhibitor
SM	DPP-III inhibitor
GY	ACE inhibitor, DPP-IV inhibitor
VQ	DPP-IV inhibitor
VK	ACE inhibitor, DPP-IV inhibitor
SF	ACE inhibitor, DPP-IV inhibitor, renin inhibitor
AY	ACE inhibitor, DPP-IV inhibitor, antioxidant
QL	DPP-IV inhibitor
QI	DPP-IV inhibitor

LQ	ACE inhibitor
IQ	DPP-IV inhibitor
LK	Antioxidant
IE	ACE inhibitor
EI	ACE inhibitor, DPP-IV inhibitor
EL	Antioxidant
SY	ACE inhibitor, DPP-IV inhibitor
LH	DPP-IV inhibitor, antioxidant
IH	DPP-IV inhibitor, DPP-III inhibitor
FN	DPP-IV inhibitor
VY	ACE inhibitor, DPP-IV inhibitor, DPP-III inhibitor, antioxidant
TY	ACE inhibitor, DPP-IV inhibitor
VGL	DPP-IV inhibitor
LR	ACE inhibitor, DPP-III inhibitor, renin inhibitor
IR	ACE inhibitor, DPP-IV inhibitor, renin inhibitor, antioxidant
FQ	ACE inhibitor, DPP-IV inhibitor
NY	ACE inhibitor, DPP-IV inhibitor
LGI	ACE inhibitor
QY	DPP-IV inhibitor
YQ	DPP-IV inhibitor
EY	ACE inhibitor, DPP-IV inhibitor
FR	ACE inhibitor, DPP-IV inhibitor, DPP-III inhibitor
YR	DPP-IV inhibitor, DPP-III inhibitor
LLP	ACE inhibitor
ILP	ACE inhibitor
LVQ	ACE inhibitor
IVQ	ACE inhibitor
VVF	ACE inhibitor
LTF	ACE inhibitor
ITF	ACE inhibitor

LEE	ACE inhibitor
LLF	ACE inhibitor
LVY	ACE inhibitor
IVY	ACE inhibitor
LLR	Antioxidant
LIY	ACE inhibitor
IIY	ACE inhibitor
LLY	Immunostimulating
IKY	ACE inhibitor
